# ModelExplorer - software for visual inspection and inconsistency correction of genome-scale metabolic reconstructions

**DOI:** 10.1186/s12859-019-2615-x

**Published:** 2019-01-28

**Authors:** Nikolay Martyushenko, Eivind Almaas

**Affiliations:** 1 Department of Biotechnology, NTNU - Norwegian University of Science and Technology, Trondheim, N-7491 Norway; 2 K.G. Jebsen Center for Genetic Epidemiology, Department of Public Health and General Practice, NTNU - Norwegian University of Science and Technology, Trondheim, N-7491 Norway

**Keywords:** Metabolic model, Network visualization, FBA, Constraint based modeling, Consistency checking

## Abstract

**Background:**

Genome-scale metabolic network reconstructions are low level chemical representations of biological organisms. These models allow the system-level investigation of metabolic phenotypes using a variety of computational approaches. The link between a metabolic network model and an organisms’ higher-level behaviour is usually found using a constraint-based analysis approach, such as FBA (Flux Balance Analysis). However, the process of model reconstruction rarely proceeds without error. Often, considerable parts of a model cannot carry flux under any condition. This is termed model inconsistency and is caused by faulty topology and/or stoichiometry of the underlying reconstructed network. While there exist several automated gap-filling tools that may solve some of the inconsistencies, much of the work still needs to be carried out manually. The common “linear list” format of writing biochemical reactions makes it difficult to intuit what is at the root of the inconsistent behaviour. Unfortunately, we have frequently observed that model builders do not correct their models past the abilities of automated tools, leaving many widely used models significantly inconsistent.

**Results:**

We have developed the software *ModelExplorer*, which main purpose is to fill this gap by providing an intuitive and visual framework that allows the user to explore and correct inconsistencies in genome-scale metabolic models. The software will automatically visualize metabolic networks as graphs with distinct separation and delineation of cellular compartments. ModelExplorer highlights reactions and species that are unable to carry flux (blocked), with several different consistency checking modes available. Our software also allows the automatic identification of neighbours and production pathways of any species or reaction. Additionally, the user may focus on any chosen inconsistent part of the model on its own. This facilitates a rapid and visual identification of reactions and species responsible for model inconsistencies. Finally, ModelExplorer lets the user freely edit, add or delete model elements, allowing straight-forward correction of discovered issues.

**Conclusion:**

Overall, ModelExplorer is currently the fastest real-time metabolic network visualization program available. It implements several consistency checking algorithms, which in combination with its set of tracking tools, gives an efficient and systematic model-correction process.

**Electronic supplementary material:**

The online version of this article (10.1186/s12859-019-2615-x) contains supplementary material, which is available to authorized users.

## Background

Genome-scale metabolic reconstructions have become a standard approach for the computational investigation of living organisms’ phenotypes [1], bridging the gap between experimental knowledge of components, such as which enzyme catalyzes which reaction, and high-level organismal behaviour (e.g. growth rate). In their simplest representation, genome-scale metabolic reconstructions only consist of chemical and transport reactions with corresponding reactants and products distributed into different compartments of an organism [2, 3].

In order to predict the growth rate of an organism from a genome-scale metabolic reconstruction, one usually applies a constraint-based modeling method such as Flux Balance Analysis (FBA). The FBA approach is based on a steady-state approximation of cellular growth. In FBA, an empirically acquired biomass function is optimized while the metabolic reconstructed network is subject to constraints on nutrient uptake reactions. Additional information may be included in the model either to further constrain it, as exemplified by the MOMENT method [4], or to expand its descriptive ability by, for instance, adding genetic regulation and protein expression explicitly as in ME models [5]. While such approaches complicate the treatment of metabolic models even further, there still does not exist a reliable automatic, or even close to automatic, workflow to create the basal metabolic reconstruction.

The draft of a high-quality genome-scale metabolic reconstruction is often created with automated tools, such as the SEED [6], or the RAVEN [7] toolboxes, that adhere to many of the proposed steps for generating a reconstruction [8]. The starting point of this procedure is commonly the annotation of an organism’s genome, which is used to elucidate what enzymes are produced and which reactions the metabolism of an organism is capable of performing. A first benchmark test of a draft reconstruction, is the assessment of its ability to produce biomass (grow) given a certain medium composition. Sometimes the metabolic reconstruction, however, is incapable of producing all the constituents of the biomass reaction on the given medium, or even on any media. Additionally, even when a draft or a published reconstructed network is capable of producing all the constituents of the biomass reaction, it is still the rule rather than the exception that the network contains reactions which are blocked in FBA simulations under any input conditions. Apart from being useless in terms of modelling, if corrected and made active, such reactions could increase the model realism and quite possibly affect phenotype predictions, such as gene essentiality, by providing alternative pathways. These generally blocked reactions result from topological and stoichiometric issues intrinsic to the model itself. Therefore their presence is termed model inconsistency, and the search for such reactions is called consistency checking.

The first-line tools in identifying and fixing the cause or causes for model inconsistencies are automatic gap-filling algorithms. These can either address all blocked reactions at once [9] or first divide them into groups called Unconnected Components, addressing each group one by one to reduce complexity [10]. Unfortunately, these tools usually cannot solve all the inconsistencies. Take as an example the Gapfind/Gapfill approach of Kumar et al. [9]: They find that up to 40% of the blocked fluxes in the *E. coli* model they were addressing could not be fixed using their algorithms, while for *S. cerevisiae* this number was 58%. This is a significant issue that must be fully acknowledged and appreciated. Existing tools are quite simply not enough. The problem is further complicated by the fact that model building and gap-filling tools may use the same metabolic reaction repositories, rendering gap-filling useless.

When automatic tools fail, it is currently necessary to manually identify the cause, or often multiple causes, for the deficits. This is a tedious process, complicated by the linear list format of metabolic models. While it is quite straightforward to identify lists of blocked reactions, using e.g. existing functions in the COBRA toolbox [11] or in COBRApy [12] framework, it is often quite challenging to identify what the inconsistencies are caused by. Usually we are talking about a small set of reactions being at the root of the problem. We have for instance observed cases when a single faulty transport reaction caused a stoichiometric lock, that effectively incapacitated a whole compartment.

The main purpose of ModelExplorer is to aid the user in correcting inconsistencies that cannot be addressed with automatic algorithms. The software provides a visual interface and multiple analysis modes to facilitate the identification of blocked reactions and in searching for and correcting the source of their inactivity. Based on our hands-on experience with manual curation of more than 10 genome-scale metabolic models, we have found that when significant parts of the metabolic network are shown to be inconsistent (for some reason, being blocked), the inconsistency can often be corrected by adding or modifying one, or very few (thus key) reactions in the network. Similarly, we identified the need for a visual workflow for model curation in order to speed up the process of fixing the large number of reactions that are not automatically corrected by current software. With ModelExplorer, the user can get an intuitive overview over every blocked part of the model, allowing the user to identify and fix key reactions which need to be corrected without leaving the software, as well as quickly identifying related, broken parts of the metabolic network.

## Implementation

ModelExplorer has been developed as a stand-alone graphical application under Linux Additional file 1 and Windows Additional file 2 and is fully written in C ++ for speed and ease of interaction with the COIN-OR Clp linear programming library [13], which is used for model consistency checking. The software uses cgraph (the C library behind Graphviz) for making metabolic network layouts, and the Allegro 5.2 gaming library for graphics. To achieve smooth graphical output also for larger networks, it uses GPU-accelerated anti-aliasing. GPU acceleration also positively affects the frame rate when moving the network in the display panel. This does not mean that the software requires a dedicated graphics card, as all modern processors possess a graphics unit. In the Windows OS, graphics drivers are usually provided out of the box. When using Linux, it is recommended to use a standalone installation of Linux (preferably Ubuntu 16.04 to 18.04) with appropriate graphics drivers enabled in order to ensure that the ModelExplorer graphics are rendered fast and smoothly. Virtual machines often do not provide direct access to the GPU. The software will take a reconstructed metabolic network in the sbml format [14, 15] as a file input.

## Results and discussion

ModelExplorer allows the visualization of a metabolic reconstructed networks as bipartite graphs: Metabolites and reactions are represented by nodes, and links (shown as arrows) only connect metabolites to reactions and vice versa. The arrows may be unidirectional or bidirectional, depending on the encoded reaction reversibility in the metabolic reconstruction. Metabolites and reactions are automatically grouped by their compartment, as specified in the reconstruction. The compartment grouping is visualized and may be highlighted.

**Fig. 1:**
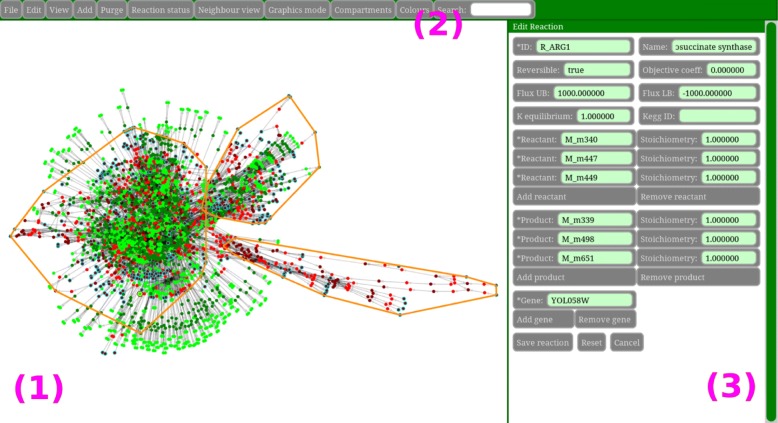
General layout of Model Explorer: The **network view** (**1**) shows a bipartite graph representation of the metabolic model, which is both zoomable and pannable. Reactions and species are represented with different shades of the same colour (reactions are bright and species are dark). If the reaction/species is active the base colour is green, and red if blocked. Endpoint species (those which are either not produced or not consumed) have a light blue outline, while the biomass (growth) reaction has a thick yellow outline. Connections between reactions and metabolites are represented with grey semitransparent arrows that have either one or two arrowheads depending on reversibility. Compartment contours are shown in orange, and all species that belong to a compartment must be localized within its contour. The **command panel** (**2**) contains a set of function menus and a Search tool, which can be used to find species and reactions by their name or id. When using neighbour view in the Command Panel, and hovering the cursor over a reaction/species or selecting it, the names of the reaction/species and a list of its properties, neighbours, ancestors or blocked module mates is shown in the **text panel** on the right (**3**)

The network layout is calculated using a force-directed algorithm, as we found this to give the most aesthetically pleasing results. In the Network View (Fig. 1, mark (1)) the user may zoom, pan, select and hover the cursor over nodes in the network in order to explore or make changes. In ModelExplorer, the user is provided with a set of options for network visualization, exploration and editing. These options may be accessed through function menus in the Command Panel (Fig. 1, mark (2)).

Some of the tools in ModelExplorer can also output information to the Text Panel (Fig. 1 mark (3)).

### Finding blocked reactions

The core of the ModelExplorer functionality is the identification of blocked reactions and metabolites that cannot be produced by a reconstructed metabolic network. This is called consistency checking. ModelExplorer provides the user with three different methods for doing this, named FBA, Bi-directional and Dynamic mode. The FBA and Bi-directional methods have previously been published in different implementations [16, 17].

In the FBA mode, a reaction is declared (and marked) blocked if it is unable to carry a (FBA) steady state flux. A metabolite is shown as blocked if all reactions that can generate it are blocked. In order to reduce the time it takes to perform consistency checking in FBA mode, we have developed a radically improved version of the FastCC algorithm [17], which we call ExtraFastCC. It uses 40-80 times fewer optimization rounds than its predecessor. Detailed speed and complexity comparisons of our algorithm against FastCC can be found in the “Comparison with other software” section.

The FBA mode is useful to identify which parts of a model may be removed without affecting the results of any FBA simulation. Restoring the consistency of these reactions may improve the model’s resilience against knock-outs. Using this mode we consistency-checked 13 models from the OpenCOBRA model repository used by Ebrahim et al. [18] (iMM1415, iAF1260, iCac802, iAN840m, iMM904, iBsu1103, iND750, iMO1056, iJN746, iJR904, iNJ661, iFF708 and iRsp1095) and found 28% of all reactions to be blocked on average, with a standard deviation of 11%. This highlights that blocked reactions as a significant problem for most metabolic reconstructions.

In the bi-directional mode, we initiate the analysis by setting all reactions to be reversible. This step is followed by running the same algorithm as used for the FBA mode. The main purpose of the bi-directional mode is not as an alternative to the FBA-mode, instead to provide the user with a quick way to check if the *inactivity* of a certain part of the model is caused by an over-constrained or misdirected reaction. In addition to help identifying obvious errors, comparing the two modes can address a deeper dilemma: It is not always trivial to establish the reversibility of a reaction, as it is influenced by the relative concentrations of the participating chemical species. Concentrations may change depending on the abundance of available nutrients, altering reaction reversibility.

Finally, in the dynamic mode, a species is declared (and marked) blocked if it will block the biomass (growth) reaction when added to the list of its reactants. A reaction is then determined to be blocked if any of its reactants are blocked. The dynamic mode is useful in the process of assessing the fidelity of a draft reconstruction, since it allows us to identify which existing metabolites may potentially be part of the biomass reaction without blocking it. It is the only mode that will show valid results when the user has not yet added a biomass function or any export reactions to the model, as the dynamic mode can solely rely on imports. This mode also adds a higher level of realism compared to the FBA mode, since it shows if the reconstructed network can support a constant concentration of a metabolite during exponential growth. Unfortunately, this topic is usually overlooked as exponential growth cannot be directly addressed using the steady state approximation of FBA. The Dynamic mode is the fastest to compute among the three modes, and it always needs only one round of optimization. The details of the algorithm will be published elsewhere [Martyushenko, Almaas. In preparation].

**Fig. 2:**
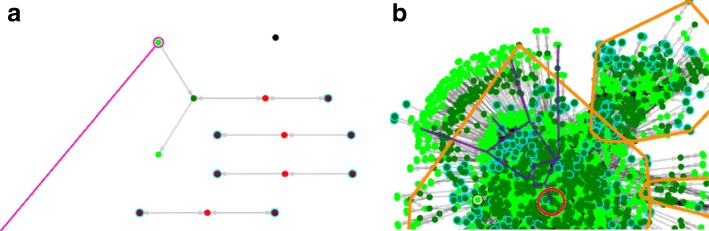
ModelExplorer graphical features: **a** The **search** tool in the command panel can be used to search for reactions and species by their id or name. If one selects the desired item from a drop-down list of matches, a purple circle is drawn around the target, and a line of similar colour is drawn from the lower left corner of the network view to the circle. **b** In node ancestry mode, one can view the shortest pathway (all the way to import reactions) necessary to produce a species or to make a reaction active. It gets highlighted in dark purple colour if non-cyclic. If cyclic, the cycle (strongly connected component) gets highlighted in black

If the user wants to know whether a specific reactions is blocked, ModelExplorer makes it is possible to directly look up reactions and metabolites by their name through the search function and highlights them on the network view (Fig. 2 panel a).

### Exploring the network

When blocked reactions and metabolites are identified, the user is presented with four tracking tools for determining the source of error (accessed through the “Neighbour view” menu).

The first option, called “None,” does not highlight anything except the node itself. However, in this mode one may edit existing species, reactions and compartments by clicking on them. By using the Text Panel, it is possible to change their properties.

The second option, called “Ego-centric,” highlights the selected node’s direct neighbours and can be used for brute force exploration of blocked nodes. For instance, it makes it easy to distinguish reactants from products, as well as to asses which reactions produce and consume a metabolite.

The third option, called “Node ancestry,” is more intricate. Here, ModelExplorer will highlight the smallest subset of the network necessary to synthesize a species or activate a reaction in question, given that a non-cyclic solution exists. One such path is highlighted by ModelExplorer in Fig. 2 panel b. If the path is cyclic, the “Node ancestry” mode will instead highlight the cycle, defined as the strongly connected component.

The fourth option is called “Blocked Module” and highlights unconnected modules, as described by Ponce-de-Leon et al. [10]. Each module is an unconnected (to other modules) group of blocked species and reactions, which can be addressed independently of other groups. This tool shows an output only when hovering over blocked items, highlighting the same module when hovering over any of its members. The “Blocked Module” tracking tool is special, because the user can choose to view the module *separately* from the rest of the network. This is done through the View menu. The layout algorithm is then run only on the blocked module, and the module is plotted on its own. This makes it much easier to visually identify the source of the inconsistency, as crowding in the visual display of the network is very much reduced. Model editing and tracking can be done on the module in the same way as on the whole model, with all changes being applied to the model itself.

### Editing the network

ModelExplorer allows the user to interactively edit, add and delete any species, reaction or compartment in the model. It can even be used to build models from scratch by hand. Editing can be performed on any object with the “None” tracking tool option activated, by right-clicking the object and then altering its properties in the Text Panel. Adding and deleting objects is done through the “Add” and “Purge” menus. In addition to deleting objects one by one, ModelExplorer provides the user with several *en masse* node purging functions. These tools may be useful if, for instance, a reconstructed network has boundary (or extracellular) metabolites instead of import reactions. In that case, ModelExplorer can purge such species, allowing reactions consuming these metabolites to become import reactions.

We have observed many publicly available reconstructed networks to consist of multiple disconnected graphs, where all graphs, except the one containing the biomass, obviously are useless from a modelling perspective. If it would be of interest to remove these, ModelExplorer includes a function to purge disconnected clusters. This function can also be useful after a purge of boundary or extracellular metabolites that may leave behind rudimentary, disconnected reactions. The user also has the choice to only purge species and reactions which are unconnected to any other species or reaction, since we have observed some models to contain unused metabolites in the hundreds.

### Comparison with other software

To our knowledge, there are at least five other packages that address the issue of visualization of metabolic networks: MetDraw [19], Escher [20], Gephi [21], Cytoscape [22] with the cy3sbml [23] plugin, and MetExploreViz [24]. None of these tools can perform or visualize consistency checking, edit the underlying model or track neighbours, ancestry or unconnected modules.

MetDraw is also based on Graphviz. However, it does not provide an interactive network view since it will only output still images. Escher and MetExploreViz, are interactive web-applications centered around pathway visualization. These tools draw networks disentangled into pathways, for which human input is necessary since the way one divides a network into pathways is strictly subjective. This approach means that side-metabolites appear plotted multiple times, which could complicate deciphering inconsistencies and tracking ancestry, if such options were to be implemented.

Cytoscape and Gephi on the other hand, are generalist network visualization tools. Cytoscape can use the cy3sbml plugin to import, layout and view SBML files, while Gephi accepts only standard graph formats such as “dot”, requiring a prior conversion from SBML into one of these formats. Both of the tools can make layouts similar to that of ModelExplorer, but lack any other functionality, as mentioned above.

This highlights a principal difference between comparable existing software and ModelExplorer: Escher, MetExploreViz, Cytoscape and Gephi are mainly designed for network visualization of finished models, whereas ModelExplorer is designed for consistency checking and correction of metabolic models at any stage of construction and refinement. This, however, does not mean that ModelExplorer is inferior at visualization. It is in fact the opposite in terms of speed. Visualization speed can be measured in terms of the frame rate, which is the number of times the image could change per second. A low frame rate slows down the navigation around the network and can be very annoying to the user. In order to conduct a reasonable a side-by-side comparison of these very different visualization tools (except MetDraw, which makes still images and thus does not have a frame rate), we tested the frame rates when visualizing the iTO977 model using a DELL laptop with an Intel Core i5-5300U CPU (see Table 1). The comparisons revealed that ModelExplorer is approximately 10.7 times faster than Cytoscape, 9.3 times faster than GePhi (given similar visualization settings), 8.4 times faster than MetExploreViz and 2.8 times faster than Escher. It is important to note that Escher was tested with a model that was about 7 times smaller than iTO977. The reason was that the Escher documentation did not recommend launching bigger models for reasons of speed, and we therefore used the largest model provided on the Escher website.

**Table 1:** Frame rate comparison of ModelExplorer with similar software, when visualizing the iTO977 model

Software	Framerate / FPS
ModelExplorer	16.0
Escher	5.7
GePhi	1.7
Cytoscape	1.5
MetExploreViz	1.9

Note that, a 7 times smaller model was used with Escher

Another field in which ModelExplorer has is a significant advance is the consistency checking algorithm we developed. Our algorithm called “ExtraFastCC” is based on the “FastCC” algorithm of Ponce-de-Leon et al. [10], but has a significantly improved ability to check the consistency of dead reversible reactions. The FastCC algorithm is used in consistency checking tools, such as PSAMM [25]. Tools, such as MC3 [26], have based their consistency checking on flux variability analysis (FVA) [16] of every reaction, which is a much slower approach. When tested against FastCC, our algorithm performs 36-80 times better in terms of the number of linear optimization problems that needs to solve, and 3-15 times better in terms of CPU time. The FastCC algorithm needs to test nearly all of the dead reversible reactions in both directions, which can be seen from the numbers in Table 2. Note that, ModelExplorer uses the relatively slow open-source optimizer Clp, while we have used FastCC from the COBRA toolbox together with state-of-the-art commercial optimizer Gurobi [27]. While our algorithm still has better computing times, the difference would be radically larger if Clp was not so slow. As this paper is focused on the visualization tool ModelExplorer, we leave the precise details of the algorithm to another paper.

**Table 2:** Run time and complexity comparisons of the ModelExplorer consistency checking algorithm “ExtraFastCC” against its predecessor “FastCC”

			FastCC		ExtraFastCC	
Model	# reacts	# rev dead reacts	# LPs	time / s	# LPs	time / s
iTO977	1536	120	215	8.0	6	0.8
iJO1366	2583	241	489	27.2	6	9.0
Recon1	3719	395	794	117.6	21	7.9

Disconnected reaction/metabolite clusters were discarded from every model before the testing in order to avoid unrealistically large LP numbers caused by running LPs on many small clusters. The first column shows the model name, the second shows the number of reactions in the model, and the third shows the number of blocked reversible reactions in the model. The numbers of reactions are recorded after disconnected-cluster purging. Note that the number of LPs used by FastCC is approximately equal to twice the number of dead reversible reactions. ExtraFastCC uses the open source solver Clp, while FastCC is run in Matlab using the much faster Gurobi [27] solver

## Conclusions

The number and complexity of genome-scale metabolic reconstructions continues to grow. For microbial reconstructed M-models, the number of reactions is in the low thousands, while microbial community reconstructions consist of tens to hundreds of thousand reactions. ModelExplorer provides the user with the ability to evaluate model quality and aids in correcting inconsistencies in models provided in the common SBML format. The visual nature of the software’s different functions makes it intuitive and easy to use, while its reliance on low level routines makes it faster than existing metabolic model visualization software.

## Availability and requirements

**Project name:** ModelExplorer v1.0.

**Project home page:** https://www.ntnu.edu/almaaslab/downloads.

**Operating systems:** Windows 8.1 and 10, Linux - Ubuntu 16.04 LTS, 17.04, 17.10, 18.04 LTS and Manjaro 17.1.1.

**Programming language:** C++

**Other requirements:** None.

**License:** Creative Commons Attribution-NonCommercial 4.0 International Licence

**Any restrictions to use by non-academics:** license needed for commercial use.

## Additional files

Additional file 1 ∙*ModelExplorer* - Linux binary file (ModelExplorer v1.0 executable)
∙*modelEpxlorerLibs* and *extraLibs* - Folders with libraries (COIN-OR Clp and Graphviz)
∙*install.sh* - a Linux installation script
∙*arial.ttf* - Font file
∙*test.xml* - Test model (iTO977)
∙*ModelExplorer_User_Manual.pdf* - User manual that contains information about software installation procedures, usage and licence.
∙*LICENSE.txt* - A license notice. (ZIP 13,984 kb)

Additional file 2 ∙*ModelExplorer* - Windows binary file (ModelExplorer v1.0 executable)
∙ A set of library files - (COIN-OR Clp and Graphviz)
∙*arial.ttf* - Font file
∙*test.xml* - Test model (iTO977)
∙*ModelExplorer_User_Manual.pdf* - User manual that contains information about software installation procedures, usage and licence.
∙*LICENSE.txt* - A license notice. (ZIP 4801 kb)

## Abbreviations

FBA: Flux balance analysis
