# Supplementary material for: ModelExplorer - software for visual inspection and inconsistency correction of genome-scale metabolic reconstructions
Source: BMC Bioinformatics. 2019 Jan 28;20:56. doi: 10.1186/s12859-019-2615-x (PMC6348647; doi:10.1186/s12859-019-2615-x)
Supplement: Supplementary file 1 — ∙ModelExplorer - Linux binary file (ModelExplorer v1.0 executable) ∙modelEpxlorerLibs and extraLibs - Folders with libraries (COIN-OR Clp and Graphviz) ∙install.sh - a Linux installation script ∙arial.ttf - Font file ∙test.xml - Test model (iTO977) ∙ModelExplorer_User_Manual.pdf - User manual that contains information about software installation procedures, usage and licence. ∙LICENSE.txt - A license notice. (ZIP 13,984 kb) [file 12859_2019_2615_MOESM1_ESM.zip › ModelExplorer/ModelExplorer_User_Manual.pdf]

ModelExplorer 1.0 User Manual

The package

ModelExplorer is shipped as an archive containing:

1) Linux or Windows binary file - “ModelExplorer”
2) Folders with libraries (COIN-OR Clp and Graphviz) - “modelEpxlorerLibs” and “extraLibs” (for Linux) or just a set of libraries (for Windows).
3) An installation script “install.sh” (on Linux only).
4) Font file - “arial.ttf”
5) A test model (iTO977) - “test.xml”
6) This manual
7) A license notice - “LICENSE.txt”

Intended use

ModelExplorer is a metabolic model visualization package that can assist the user in finding blocked parts of the metabolic network as well as finding out why they are blocked. For this, the user is provided with three different definitions of inactive reactions: *FBA* and *Bidirectional* for correcting finished models, and *Dynamic* for building new ones. ModelExplorer assists the user in finding the source of network blocking with two tracking tools – one which shows the node’s nearest neighbours, and one that shows a minimal reaction path necessary to produce a metabolite. Blocked reactions and non-produced metabolites can often be the result of faulty transport between compartments. The network layout algorithm used in ModelExplorer therefore visually separates and highlights different cellular compartments, in order to make troubleshooting easier and more intuitive. Finally ModelExplorer has all the necessary tools required to edit existing models and build up new ones by hand.

Installation

The program comes in two versions: Windows and Linux. The Windows version has been tested to run on Windows 10, while the Linux verision has been tested to run on Ubuntu 17.04, 17.10, 16.04 LTS and 18.04 LTS, as well as on Manjaro 17.1.1. We recommend using a true installation of the system and not a virtual machine, as the latter will likely not be able to take advantage of the GPU.

On Windows no installation is required. The only premise is that ModelExplorer executable is kept in the same folder as the libraries and the font file. The user is advised to place the content of the archive into a single folder and make a shortcut to the ModelExplorer executable. Alternatively the program can be run from a terminal (cmd) window, which is advisable in case troubleshooting will be required.

The Linux installation procedure works as follows:
Extract the archive to a folder of your choice. Run the installation script, agreeing to everything that the user will be prompted with (it will preform a system update and install the required libraries, copying the libraries shipped with ModelExplorer into a system folder).

```
sudo ./install.sh
```

After the installation script has been run, ModelExplorer can be launched from within a terminal or directly. Running it from terminal is advisable in case troubleshooting will be required.

Launching

The program should be run from the terminal and the command could be followed by a path to a model file that is to be opened. For example on Linux:

```
./ModelExplorer test.xml
```

Or on Windows:

```
ModelExplorer.exe test.xml
```

The program accepts only **SBML2** as the model format. A model could be loaded either with the command above or from within the GUI. If reactions refer to undefined metabolites, undefined compartments or lack stoichiometries, the user will be presented with self-explanatory warnings. If species, reactions or compartments lack the “id” field, an appropriate error message will be presented, and the model will not be opened. When the model gets loaded the user is presented (in the terminal) with model name, flux unit and the number of chemical species and reactions in the model:

```
/////////////////////////////////////////////////////////////////////////////////////////////
//                                                                                         //
//   Opened Model:            Flux Unit:              Species Number:         Reaction Number:   //
//   iTO977                   mmol_per_gDW_per_hr     1218                    1560              //
//                                                                                         //
/////////////////////////////////////////////////////////////////////////////////////////////
```

When a model is loaded, the program will immediately proceed to calculating which metabolites and reactions are blocked in the three available blocking modes. This and the following graphical layout procedure may take some time, especially for models with more than 3000 reactions / metabolites. *For certain models with a lot of metabolites that are not utilized in any reaction (e.g. Recon2), it is recommended to use the*
“-ps”
flag before the path to the model in order to remove these species and decrease the time it takes to make a layout of the model. In case of very large models (with connected parts >10^4 *reactions) it may take a long time to run all but the Dynamic blocked reaction algorithm, so the user can use*
“-nocalc”
flag to save time and only do the Dynamic calculations.

Graphical User Interface:

When the model is loaded the user is presented with the GUI of ModelExplorer. On top is the ***command panel*** with all available commands (1), to the left is the ***network view*** in which the model is visualized (2), and to the right is a ***text panel*** (3), which is used to output information from the neighbour viewing tools and input/output information from model editing tools.

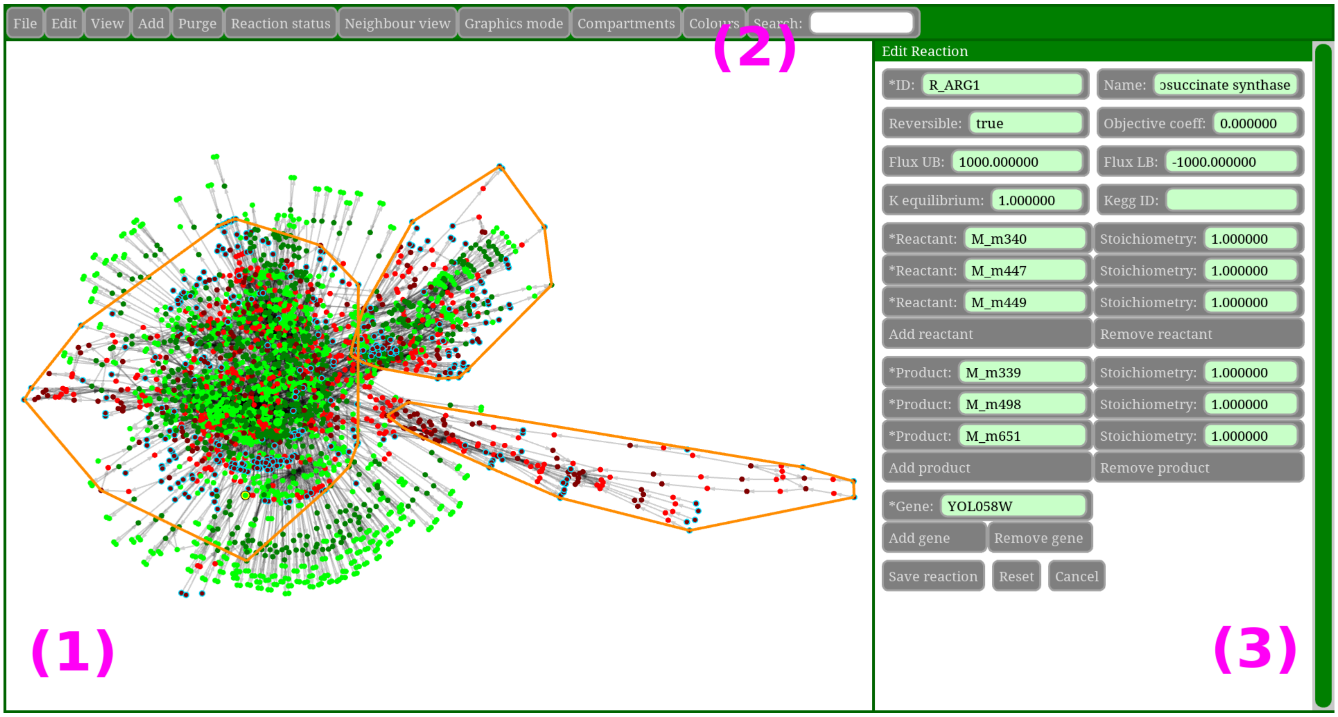

Command Panel:

The panel contains a set of function menus and a ***Search*** tool. The search tool can be used to search reactions and species by their id or name, by typing a part of that id or name (not shorter than 2 letters) and selecting one of the suggestions that appear. The search tool is ***case sensitive***. When an entry is selected **purple** circle is drawn around the target, and a line of similar colour is drawn from the lower left corner to the circle:

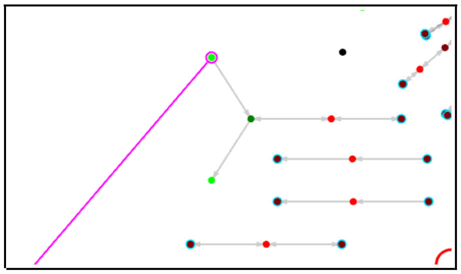

The set of menus is as follows:

1) **File** – make ***new***, ***open*** and ***save*** models, ***quit*** the program. ***Save as SVG*** saves the current network view to a file in SVG format (vector graphics). ***Save blocked*** saves the blocked reactions and species found in the three modes (*FBA*, *Bidirectional and Dynamic*) into CSV files, grouped into columns by their blocked module, sorted by module size (descending).

2) **Edit** – ***undo*** and ***redo*** the last change to the model (adding, removing or changing species, reactions and compartments), ***replot*** and ***recalc***ulate blocked reactions/species.
3) **View** – ***Blocked module*** and ***Whole model*** plots only the selected blocked module or the whole model respectively. The former mode is to reduce complexity when resolving inconsistencies.
4) **Add** – ***species***, ***reaction***, ***compartment*** to the model (for model editing and building). The user gets to fill out a set of input boxes in order to create a new species, reaction or node. The user can click the "Save species/reaction/compartment" of "Cancel" buttons in order to add a new entry or close the adding menu. Entries marked with an asterisk (*) are obligatory to fill out. The rest are not, but rules can apply to the content. The sets of entries are:
   Add ***species:***
   - ID – required, must be unique
   - Name – not required, can by anything
   - Compartment – not required, must select one of existing compartments or none
   - Boundary condition – required, must be either true or false
   - Formula – not required, can be anything
   - Kegg ID – not required, can by anything

   Add ***reaction***:
   - ID – required, must be unique
   - Name – not required, can by anything
   - Reversible – required, can be either true or false
   - Objective coeff (coefficient) – not required, must be a number
   - Flux UB (upper bound) – not required, must be a number
   - Flux LB (lower bound) – not required, must be a number
   - K equilibrium (equilibrium constant) – not required, must be a number
   - Kegg ID – not required, can by anything
   - Add Reactant – button adds reactant to the end of the list
     - Reactant – required, must select one of existing species
     - Stoichiometry – required, must be a number (set to 1 by default)
   - Remove Reactant – button removes last reactant in list
   - Add Product – button adds product to the end of the list
     - Product – required, must select one of existing species
     - Stoichiometry – required, must be a number (set to 1 by default)
   - Remove Product – button removes last product in list
   - Add Gene – button adds gene to the list of genes
     - Gene – required, specifies an identifier of the generate
   - Remove Gene – button removes last gene in list

   Add ***compartment***:
   - ID – required, must be unique
   - Name – not required, can by anything
   - Outside – not required, (compartment outside the current compartment), must select one of existing compartments or none

   An input box gets red if the input is rejected and green if it is accepted. If all entries are valid and the user clicks the "Save" button, a new species gets placed at a random position on the screen or a new reaction gets placed at the barycenter of its reactants and products or a compartment gets created (but does not appear until species are added to it).
5) **Purge** – remove the selected option:
   - ***Selection*** – removes a species, reaction or compartment selected with a RMB click.
   - ***Boundary species*** (names ending in "_b" or "_boundary").
   - ***Extracellular species*** (names ending in "_e" or "_extracellular"). This and the previous options were added in order to correct some models in which cellular import was initiated in a boundary or extracellular species instead of a reaction which only has products.
   - ***Disconnected species*** (not participating in any reaction).
   - ***Disconnected reactions*** (lack both reactants and products).

- ***Disconnected clusters*** (remove any clusters (as well as singlets) of reactions and species not connected to the largest cluster).

6) **Reaction status** – visualize blocked reactions and species according to:
   - ***Always on*** – no reactions or species are shown as blocked.
   - ***FBA*** – a reaction is shown blocked if it cannot carry a steady state flux. A species is shown as blocked if all reactions that can generate it are blocked.
   - ***Bi-directional*** – setting all reactions to be bidirectional, a reaction is shown as blocked if it cannot carry a steady state flux. A species is shown as blocked if all reactions that can generate it are blocked.
   - ***Dynamic*** – a species is shown as blocked if it will block the biomass (growth) reaction when added to it. Useful in the process of building models, as it shows if the cell can produce that species dynamically or not. A reaction is blocked if any of its reactants is blocked.
7) **Neighbour view** – highlight the species/reactions linked to the currently active one. A reaction/species is made active by holding the cursor over its node in the network:
   - ***None*** – show only the current species/reaction and an ***Edit menu*** for it (in the Text Panel).
   - ***Ego-centric*** – if the active node is a reaction, show its reactants and products. If the active node is a species, show producing and consuming reactions.
   - ***Node ancestry*** – track the shortest path (all the way to import reactions) necessary to produce a species or to make a reaction active. Visualized in **purple** colour. Possible only if the pathway is non-cyclic (is not a part of a strongly connected component). If cyclic, the cycle (strongly connected component) is visualized in **black**. Strongly connected components may constitute large parts of a model.
   - ***Blocked module*** – if a species or reaction is blocked, shows the connected blocked module (the largest possible set of connected blocked reactions and species). This module can be viewed for itself using ***View*** → ***Blocked module*** in order to make model correcting easier.
8) **Graphics mode** – determine the resolution of the arrows linking the nodes (the most graphically intense part of drawing):
   - ***High resolution*** – full resolution.
   - ***Low resolution*** – half (in each direction) of the full resolution.
9) **Compartments** – ***Show*** or ***Hide*** the visualizations of cellular compartments.
10) **Colours** – set the colours of:
    - ***Active reactions'*** nodes
    - ***Active species'*** nodes
    - ***Blocked reactions'*** nodes
    - ***Blocked species'*** nodes
    - Contours around ***Endpoint species*** (species that lack either producing or consuming reactions).
    - ***Disjoint species'*** nodes (species that are not associated with any reactions).
    - Contours around cellular ***Compartments*** (as well as the text colour that tells which compartment the cursor is currently inside).

Network view:

The network view shows a bipartite graph representation of the metabolic model. The nodes are connected by ***gray*** semitransparent arrows. If a reaction is unidirectional, the arrows have only one arrowhead (indicating the reaction direction). If bidirectional, the arrow has two arrowheads. Reactions and species are represented with different shades of the same colour (reactions are ***bright*** and species

are ***dark***). If the reaction/species is active the base colour is ***green***, and ***red*** if blocked. Endpoint species (those which are either not produced or not consumed) have a ***light blue*** outline, while the biomass (growth) reaction has thick ***yellow*** outline.

When the user hovers the cursor over a species or reaction, information about it is displayed in **purple** in the upper left corner of the view. This information contains the ***name*** of the reaction/species, its ***id*** in [square] brackets and if species, its parent ***compartment*** in (round) brackets. When Neighbour View is set to ***None***, an edit menu appears in the text panel to the right, showing model-related information about the species, reaction or compartment (the same as when you add a new one). If the user right clicks a species, reaction or compartment, the ***edit menu*** gets fixed on it, so the user can make changes to it. Colours can be changed using the panel *Colour* menu.

Cellular compartments are represented with ***orange*** outlines. Each outline contains all the species in that compartment, but may also contain species from other compartment (if compartments intersect). The drawing of compartment outlines can be turned off using the *Compartments* menu in the panel. Even when turned off, the name of the compartment in which the cursor currently is will be displayed in ***orange*** in the upper left corner of the view below the species/reaction information. This colour can also be changed.

Network view – neighbour view:

The ***Neighbour view*** option in the panel allows the user to choose between *Ego-centric*, *Node ancestry* and *Blocked module* modes. In the ego-centric mode, when the user holds the cursor over a species or reaction the links to its direct neighbours are highlighted. For reactions these are ***red*** for reactant and ***blue*** for products (left picture). For species these are ***red*** for producing, ***blue*** for consuming and ***purple*** for bidirectional reactions (right picture).

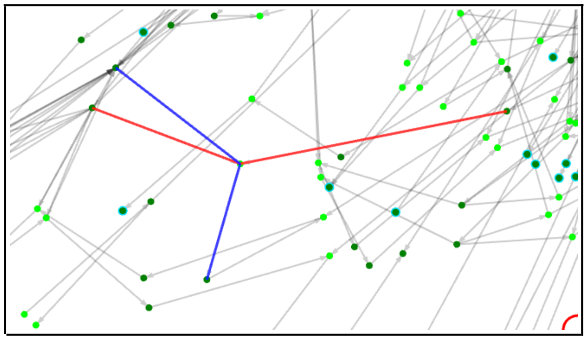

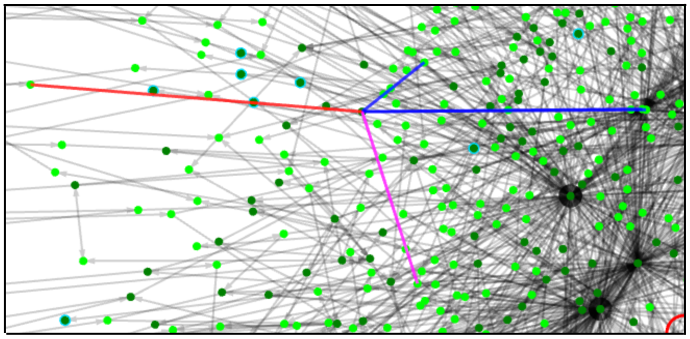

One can freeze the current selection by right-clicking the node (and continue viewing neighbours of other nodes while the ones of the frozen are still shown), and unfreeze by clicking elsewhere.

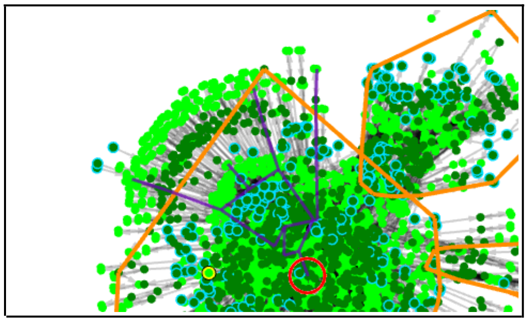

In the node ancestry mode, the one can view the shortest pathway (all the way to import reactions) necessary to produce a species or to make a reaction active (see picture below). The pathway is shown in **purple** colour. It is possible to draw this pathway only if the pathway is non-cyclic (is not a part of a strongly connected component). If cyclic, the cycle (strongly connected component) is visualized in **black**.

In the Blocked module mode, ModelExplorer highlights in **black** the set of connected blocked reactions and metabolites to which

the target species or reaction belongs (i.e. the blocked module). The module can be viewed on its own using ***View*** → ***Blocked module***, in order to make the search for a possible cause of the inconsistency in this blocked module less complicated.

Text panel:

When Neighbour View is set to ***None*** and the user hovers the cursor over or clicks a species, reaction or compartment, the text panel shows an ***Edit menu*** for this object. When hovering, the edit menu can only be used for viewing information about the object. When an object is selected (with the RMB), the edit menu can be used to change its properties.

When using the Neighbour View menu as discussed above, the name, [id] and (compartment) is diplayed for all the plotted species and reaction in the text panel. In the ego-centric mode, one sees a list of producing, consuming and bidirectional reactions (left picture below). In the node ancestry mode one can see list of the node's ancestor nodes by "generation" - the shortest distance to the node (see right picture below).

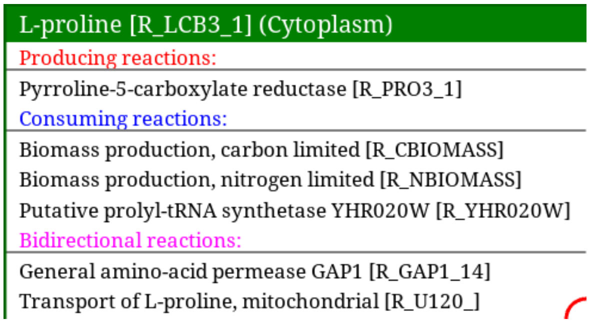

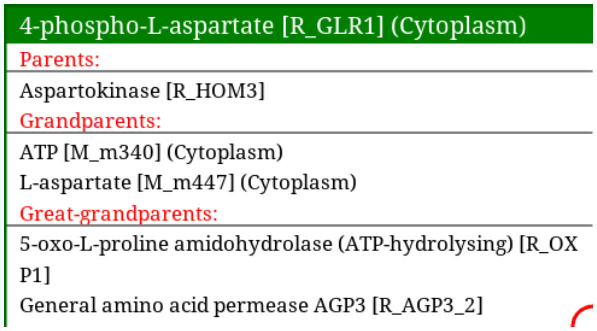

License

The software is provided as a binary executable under the Creative Commons Attribution-NonCommercial 4.0 International License, a copy of which can be found in LICENCE.txt shipped with this manual.
